# Differential gut microbiome composition in three-spined stickleback populations with contrasting levels of mercury accumulation

**DOI:** 10.3389/fmicb.2025.1673354

**Published:** 2026-01-07

**Authors:** Marijn Kuizenga, Aruna M. Shankregowda, Prabhugouda Siriyappagouder, Vyshal Delahaut, Federico C. F. Calboli, Lieven Bervoets, Brijesh Singh Yadav, Filip A. M. Volckaert, Gudrun De Boeck, Joost A. M. Raeymaekers

**Affiliations:** 1 Faculty of Biosciences and Aquaculture, Nord University, Bodø, Norway; 2 Department of Biosciences, Swansea University, Swansea, United Kingdom; 3 ECOSPHERE, Department of Biology, University of Antwerp, Antwerp, Belgium; 4 Laboratory of Biodiversity and Evolutionary Genomics, KU Leuven, Leuven, Belgium; 5 Natural Resources Institute Finland (Luke), Helsinki, Finland

**Keywords:** ecotoxicology, gut microbiome, mercury, microbiota, pollution, river, three-spined stickleback

## Abstract

**Introduction:**

Environmental micropollutants and other anthropogenic xenobiotics are potential drivers behind compositional shifts and functional dysregulation of gut microbial communities. Mercury and many of its compounds are highly toxic and ubiquitous environmental pollutants that pose a risk for aquatic biota and humans. Here we compared the gut microbial communities of natural three-spined stickleback (*Gasterosteus aculeatus* Linnaeus, 1758) populations in Flanders, Belgium, with contrasting muscle mercury concentrations. We hypothesized that exposure to a high mercury load selects for gut flora species with the capacity to tolerate or adapt to this stressor and, thus, leads to a change in the composition of the gut microbiota.

**Methods:**

The gut microbiota of 128 host individuals from four populations with low levels of accumulated mercury and four populations with high mercury levels were characterized using 16S rRNA amplicon sequencing. Gut microbial communities were compared across host muscle mercury content levels, host populations and sexes to consider the contribution of these factors in the observed differences in gut microbial diversity and composition.

**Results:**

Microbial community composition varied significantly between males and females, as well as between host populations with high and low muscle mercury content. While the abundance of 22 amplicon sequence variants (ASVs) was associated with the host’s muscle mercury content, we detected no specific indicator species for high mercury.

**Conclusion:**

Overall, our results suggest that local factors specific to a host population, potentially including mercury accumulation and sex-specific factors, differentiate the microbial communities inhabiting the gastrointestinal tracts of the three-spined stickleback.

## 1 Introduction

The widespread emission of anthropogenic pollutants has become a major contributor to global ecosystem disturbance, defaunation, and biodiversity loss (Young et al., 2016). Aquatic biota are particularly at risk for the impacts of pollution since the water they inhabit and respire can retain high quantities of contaminants in solution and suspension (Young et al., 2016). Mercury and its compounds (hereafter “mercury”) have received particular attention in this context due to their global ecotoxicological signature and environmental persistence (Selin, 2009; UN Environment, 2019). They are the only group of metallic compounds on the European Union’s (EU) Water Framework Directive’s (WFD) list of priority hazardous substances with a separate Environmental Quality Standard (EQS) for biota (European Commission, 2013; Teunen et al., 2017). Mercury acts primarily as a neurotoxin, eliciting neurotoxicity and tissue damage through disruption of neuronal functioning and calcium homeostasis, stimulation of neural excitotoxins, and interference with neurotransmission (Bernhoft, 2012; Castoldi et al., 2003; Clarkson and Magos, 2006; Rice et al., 2014). Moreover, mercury toxicosis can cause other impairments such as oxidative stress, kidney damage, endocrine disruption, and immunotoxicity (Bernhoft, 2012; Clarkson and Magos, 2006; Rice et al., 2014; Scheuhammer et al., 2007; Sevcikova et al., 2011). These toxic effects in combination with mercury’s tendencies to transform, bioaccumulate, and biomagnify, enable mercury to spread through aquatic food webs, ultimately threatening the piscivorous fishes commonly used for human consumption (EFSA Panel on Contaminants in the Food Chain (CONTAM), 2012).

A growing body of evidence suggests that symbiotic microbiota may play a role in the responses of aquatic organisms to pollution (e.g., Bertucci et al., 2022; Gaulke et al., 2016; Hacioglu and Tosunoglu, 2014). Indeed, the gastrointestinal tract and its symbiotic microbiota appear to play key roles in uptake, excretion, and toxicity following dietary mercury exposure (Johnston et al., 2022; Yang et al., 2021; Yin et al., 2023; Lin et al., 2020; Tan et al., 2022). Mercury exposures at environmentally relevant concentrations affect the composition of the gut microbiome in ways which have been linked to gut dysbiosis in both terrestrial and aquatic animals (Nielsen et al., 2018; Zhao et al., 2020; Watson et al., 2021). Additionally, the microbial biotransformation of mercury in the intestines may play a crucial role in both the reabsorption and depuration of mercury (Li et al., 2019; Tan et al., 2022).

Studies on gut microbiota in three-spined stickleback (*Gasterosteus aculeatus* Linnaeus, 1758) have previously identified associations between microbial community composition and various abiotic, biotic, and host-specific factors (Bolnick et al., 2014; Fuess et al., 2021; Hahn et al., 2022; Smith et al., 2015; Rennison et al., 2019). Three-spined sticklebacks are also an increasingly popular bioindicator and sentinel organism for ecotoxicological studies (e.g., Brosens et al., 2015; Catteau et al., 2021; Katsiadaki et al., 2002; Pottinger et al., 2002; Uren Webster et al., 2017). Yet few studies have, to our knowledge, investigated associations between stickleback gut microbial community composition and pollutant exposure. Considering the involvement of the intestinal tract and its symbionts, comparing the gut microbiota of natural stickleback populations with differing levels of accumulated mercury can provide novel insights into the metaorganism-level response to mercury.

In Flanders (Belgium), historical small-scale industrial activity, including felt production and non-ferro metallurgical industries have tainted the riverscape with a scattered signature of metal pollution (Tack et al., 2005). Consequently, mercury levels in sediments and aquatic biota remain locally elevated and occasionally exceed the EU’s EQS for biota (Bonnineau et al., 2016; European Commission, 2013; Teunen et al., 2017). Similarly, mercury concentrations in the muscle tissue of local stickleback populations vary considerably (ranging from 22.0 to 326.1 μg.kg^−1^ dry weight (DW); Calboli et al., 2021). Thanks to these inter-population variations, the Flemish riverscape has proven to be a fruitful system for describing responses to long-term sub-lethal mercury exposure in freshwater fish populations (e.g., Bonnineau et al., 2016; Maes et al., 2013; Teunen et al., 2017). Calboli et al. (2021), for instance, identified specific genomic regions on chromosome 4 of three-spined stickleback that were significantly associated with the variation in mercury concentration, suggesting a genetic response to mercury-polluted environments. However, inter-population differences in gut microbiota-facilitated plasticity have thus far remained uninvestigated in this study system and could offer valuable additional insights into the variable ecological impacts of and biological responses to mercury pollution.

Here we investigate the effects of mercury accumulation on wild three-spined stickleback populations by studying the diversity and composition of their gut microbiota. Considering the involvement of the gut and its microbiota in dietary mercury exposures in other fishes, we postulate that exposure to high mercury loads selects for gut flora species with the capacity to tolerate or adapt to mercury. Consequently we expect to observe a change in bacterial composition associated with contamination-driven perturbations of the gut ecosystem. To test this expectation, we compared the gut microbiota of individuals from four host populations with a high median muscle mercury content to those from four host populations with a low median muscle mercury content. Additionally, we compared the gut microbiota across host populations and sexes to consider the contributions of other factors. In doing so, this study aims to enhance our understanding of the role of host-microbial symbiosis in response to anthropogenic stressors and polluted environments.

## 2 Methods

### 2.1 Study area, stickleback collection, and processing

Three-spined stickleback individuals were collected from locations at eight rivers and streams across the lower catchments of the Maas River and the eastern and western basins of the Scheldt River in Flanders, Belgium. The locations represent a subset made *a posteriori* from a sampling and tissue collection effort of 21 sites described extensively in Calboli et al. (2021) (Supplementary Table S1). Briefly, 25 three-spined stickleback individuals were collected at each site using dip nets (mesh size: 5 mm) during September–October of 2017 under a permit granted by the Flemish Agency for Nature and Forest (ANB). After collection, the fish were kept in oxygenated buckets containing 25 L of water from their respective sampling sites and transported to the laboratory facilities at the University of Antwerp (Antwerp, Belgium). There, the individuals were starved overnight under continuous aeration and subsequently sacrificed with an overdose of neutral buffered Tricaine-mesylate (MS-222, Sigma Chemicals) with procedural approval from the Ethical Commission Animal Experiments of KU Leuven.

The fish were dissected on a glass plate on ice to preserve tissues. The sex of the individuals was recorded during dissection if their gonads were mature or afterwards by genotyping (see Calboli et al., 2021). For mercury analysis, the muscle tissue between the caudal end of the dorsal spines and the caudal peduncle was dissected bilaterally (75 mg of tissue on average). After removal of the skin, the muscle tissue was snap-frozen in liquid nitrogen and stored at −80 °C until analysis. For the current gut microbiome analysis, the whole intestinal tract and gut contents of each fish were removed and transferred to 2 mL sterile polypropylene cryovials with RNA*later*® (ThermoFisher Scientific, Waltham, MA, United States) prior to long-term storage at −80 °C.

### 2.2 Mercury analysis

Quantification of the total mercury content (Hg) in the muscle tissue of the collected individuals is described in detail in Calboli et al. (2021). In short, the muscle tissue samples were vacuum freeze-dried for 48 h at −55 °C and prepared for metal analysis following a protocol by Verhaert et al. (2019). Subsequent metal analysis was conducted using a high-resolution inductively coupled plasma-mass spectrometer (HR-ICP-MS, Thermo Scientific Finnigan element 2, United States). The total combined inorganic and organic mercury content was converted to ng.g^−1^ DW. Mean and median mercury concentrations of all 21 locations, as reported by Calboli et al. (2021), are presented in Supplementary Table S1.

### 2.3 Mercury-based selection of study locations

**Figure 1:**
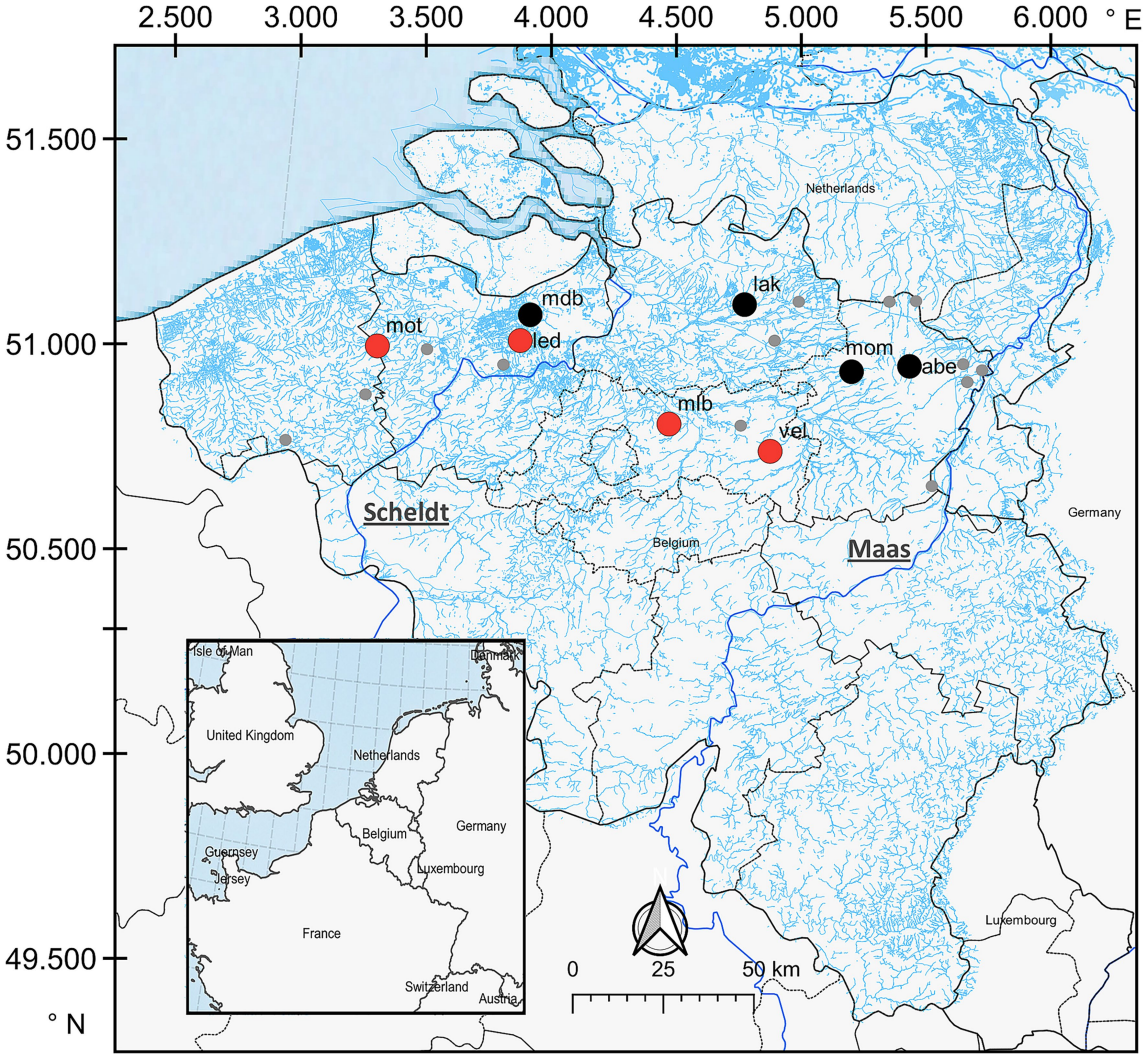
Map of the sampling region with the locations of the 21 study populations described in Calboli et al. (2021) indicated in grey, red, and black. For the present study, four locations marked in red with the highest median mercury concentrations (VEL: 195.71 ng·g^−1^; MOT: 218.23 ng·g^−1^; LED: 302.70 ng·g^−1^; and MLB: 326.634 ng·g^−1^) and four marked in black with the lowest median mercury concentrations (ABE: 21.56 ng·g^−1^; MDB: 45.13 ng·g^−1^; MOM: 47.5 ng·g^−1^; and LAK: 50.98 ng·g^−1^) were selected. These eight sampling locations are identified by a unique three letter code based on the name of the location (see Supplementary Table S1). The main course of the Scheldt and Maas are marked in blue.

Based on the mercury concentrations in the muscle tissue (Supplementary Table S1), a sub-selection of eight stickleback host populations from four sites with the lowest median muscle mercury content (Abeek [abe]: 21.56 ng.g^−1^; Molenaarsdreefbeek [mdb]: 45.13 ng.g^−1^; Mombeek [mom]: 47.5 ng.g^−1^; Laakbeek [lak]: 50.98 ng.g^−1^) and four sites with the highest median muscle mercury content (Velpe [vel]: 195.71 ng.g^−1^; Motebeek [mot]: 218.23 ng.g^−1^; Lede [led]: 302.70 ng.g^−1^; Molenbeek [mlb]: 326.63 ng.g^−1^) was made for the current study (Figure 1; Supplementary Table S1; all values presented as dry weight concentrations). Assuming a typical moisture content of 80% in fish tissue, these median dry weight concentrations correspond to 4 to 10 ng.g^−1^ wet weight for the low mercury populations and 39 to 65 ng.g^−1^ wet weight for the high mercury populations. The high mercury populations are thus exceeding the European Biota Quality Standard of 20 ng.g^−1^ wet weight (European Commission, 2013). With three locations from Scheldt-W (*mot*, *led* and *mdb*), four locations from Scheldt-E (*mlb*, *mom*, *vel*, *lak*), and one location from the Maas basin (abe), we ensured that there was no geographic clustering between populations of the high mercury and low mercury group. Lastly, to reduce potential heterogeneity in the developmental stages of the fish, individuals with a similar body size (ca. 3–5 cm in standard length) were chosen for downstream analysis.

### 2.4 DNA extraction, amplicon DNA library construction, and sequencing

The frozen intestinal tissue samples from the eight host populations were shipped on dry ice from Antwerp University (Antwerp, Belgium) to Mørkvedbukta Research Station (Nord University, Bodø, Norway), and were stored at −80 °C upon arrival. A further sub-selection of 128 randomly picked individuals (16 individuals per population) was made prior to microbiome analysis.

The intestinal tract of each individual was allowed to thaw at room temperature before drying off any RNA*later*® residue with a clean paper towel. The tissue was fragmented using sterilized scissors before adding it to a 2 mL centrifuge tube containing glass beads (~ 0.3 mL each of 0.5 mm and 0.1 mm beads; Scientific Industries Inc., Bohemia, NY, United States) and 900 μL of inhibitX buffer (Qiagen GmbH, Germany). Three rounds of homogenization were conducted at 6,000 rpm for 3 × 60 s using a Precellys tissue homogenizer (Bertin Technologies SAS, France), followed by centrifugation for 3 min at 20,000 × g after each round. DNA was extracted from the homogenate using the QIAmp® Fast DNA Stool Mini Kit (Qiagen GmbH, Germany). The quality and quantity of the total DNA extract were assessed with microvolume UV spectrophotometry and fluorometry using the NanoDrop OneC system and the Qubit® system with the Qubit® dsDNA HS assay kit, respectively (ThermoFisher Scientific).

PCR amplification was conducted using bacteria-specific Illumina-compatible primers targeting a ~ 460 bp section of the prokaryotic 16S rRNA gene between 314F (5’-CCTACGGGNGGCWGCAG-3′) and 805R (5’-GACTACNVGGGTWTCTAATCC-3′) spanning the hypervariable V3-V4 regions (Klindworth et al., 2013). All amplicon PCR reactions were performed using a thermocycler (Bio-Rad Laboratories, Inc., Hercules, CA, United States) with a 25 μL reaction volume consisting of 12.5 μL AmpliTaq gold 360 Master Mix (Thermo Fisher Scientific), 1 μL (10 μM) of each primer, and 30 ng of DNA template using the following thermocycling conditions: initial denaturation at 95 °C for 10 min, followed by 35 cycles of 95 °C for 30 s, 56 °C for 30 s, and 72 °C for 1 min, and a final step elongation at 72 °C for 7 min (Siriyappagouder et al., 2018). The amplified product was subsequently purified by separating the PCR product on a 1.5% w/v agarose gel followed by excision of positive bands (~550 bp) and gel purification using the QIAquick® Gel Extraction Kit (Qiagen GmbH) following the manufacturer’s protocol.

To obtain the final amplicon libraries, the purified PCR product was used as a template for index PCR using Nextera XT Index primers (Illumina Inc., San Diego, CA, United States), followed by clean-up using Mag-Bind TotalPure NGS magnetic beads (sample to beads ratio = 1: 1.12; Omega Bio-tek, United States). Index PCR reactions were performed with a 25 μL reaction volume consisting of 12.5 μL AmpliTaq gold 360 Master Mix (Thermo Fisher Scientific), 1 μL (10 μM) of each primer, 8 μL of nuclease-free water, and 2.5 μL of the template using the following conditions: initial denaturation at 95 °C for 3 min, followed by 35 cycles of 95 °C for 30 s, 55 °C for 30 s, and 72 °C for 30 s, and a final elongation step at 72 °C for 5 min. The DNA concentrations of the 128 indexed amplicon libraries were then quantified on the Qubit4 system. Additionally, DNA concentrations and amplicon length of 3 randomly selected libraries per host population were cross-checked by automated electrophoresis on the 2200 TapeStation® system (Agilent Technologies, Santa Clara, CA, United States). Then, the DNA libraries were pooled to equimolar concentrations of 5 nM. Lastly, the final DNA concentrations, fragment size distribution, and quality of the pooled libraries were assessed on the 4150 TapeStation® system (Agilent Technologies), followed by a final quantification using the KAPA Library quantification kit (Roche, Basel, Switzerland) and the Qubit® dsDNA HS assay kit (Thermo Fisher Scientific). Paired-end (300 bp) sequencing of the DNA libraries was performed at the Norwegian Sequencing Centre (Oslo, Norway) using the Illumina Miseq® platform with a MiSeq® reagent kit (Illumina Inc.).

### 2.5 Bioinformatic analyses

The raw paired-end reads were demultiplexed and assigned to their corresponding sample IDs at the Norwegian Sequencing Centre. Initial sequence quality was also assessed and visualized using fastQC and summarized with multiQC (Andrews, 2010; Ewels et al., 2016). Subsequent adapter removal, quality trimming, de-noising, and merging of the reads were performed using the DADA2 plugin v2022.11.2 built into the QIIME2 toolkit v2022.11.1 with the following settings: --p-trim-left-f 13, −-p-trim-left-r 13, −-p-trunc-len-f 240, and --p-trunc-len-r 240 (Bolyen et al., 2019; Callahan et al., 2016). Identification and taxonomic classification of Amplicon Sequence Variants (ASVs) were conducted with QIIME2’s feature-classifier function using a naïve Bayes machine-learning classifier specific to our 314F/805R primers (Bokulich et al., 2018; Pedregosa et al., 2011). The SILVA ribosomal RNA sequence database v138.1 SSURef NR99 was used as the reference for training the classifier and for subsequent taxonomic assignment of ASVs (Pruesse et al., 2007; Quast et al., 2013; Rognes et al., 2016).

The resulting ASVs were then filtered for singletons, unassigned ASVs, and ASVs assigned to Archaea, Euryarchaeota, mitochondria, and chloroplasts. Then, only samples with a minimum of 5 different ASVs per sample were selected for downstream analyses. Rarefaction curves generated by the alpha-rarefaction function in QIIME2 were used to select an appropriate sampling depth (i.e., number of reads). Differences in the number of reads between samples were then normalized by repeatedly rarefying to an equal sampling depth of 9,500 reads for 10,000 iterations using the repeat-rarefy v1.0 plug-in in QIIME2 (Xia, 2021). This iterative rarefaction approach was chosen to address several known limitations of traditional rarefaction (Cameron et al., 2021; McMurdie and Holmes, 2014). QIIME2 artifacts were imported into R and merged into a phyloseq object using the *qiime2R* v0.99 package (Bisanz, 2018). All downstream analyses were conducted using the R v4.2.2 language in the Rstudio environment v2022.12.0 + 353 (R Core Team, 2021; RStudio Team, 2021).

### 2.6 Statistical analyses

Statistical data analyses were conducted using the functionality of the *phyloseq* v1.42.0 (McMurdie and Holmes, 2013) and *vegan* v2.6–6 (Oksanen et al., 2022) packages, aided by functions from *ggplot2* v3.4.1 (Wickham, 2016) for data visualization and functions from base R and *tidyverse* v2.0.0 (Wickham et al., 2019) for data management and transformations. In doing so, we aimed to (1) identify the shared and unique gut microbiota of the four three-spined stickleback populations with a high mercury content and the four populations with a low mercury content, and (2) characterize potential population-specific and sex-specific differences in the composition of the gut microbiota in the host. We therefore compared both the alpha- and beta diversity of the stickleback gut microbiota across the eight host populations. Because some analyses required complete records, five individuals with unknown sex were randomly assigned to males or females. To account for any bias this may have introduced, all analyses were also run without these five individuals, and their inclusion/removal was found not to affect any downstream statistical inferences. These individuals were therefore kept in the dataset.

#### 2.6.1 Taxon composition

First, a Venn diagram was made using the *ggplot2*-compatible *ggvenn* v0.1.9 package (Yan, 2023) to assess the overall distribution of ASVs between male and female individuals and individuals with low and high mercury content. Second, the 10 most abundant phyla and 30 most abundant genera were aggregated by host population (and for phyla also by mercury content and sex) using the *microbiome* v1.20.0 package (Lahti and Shetty, 2019), and their relative abundances were visualized.

To identify potential gut microbial indicators for a high or low mercury content status, indicator values of ASVs were calculated by Dufrene-Legendre indicator species analysis based on their relative abundance and group fidelity (Dufrene and Legendre, 1997). Indicators were defined as ASVs with an indicator value *d* > 0.5 and a *p*-value < 0.01.

Lastly, to identify taxa with differential abundance between populations with high and low mercury content, we converted a phyloseq object containing the non-rarefied abundance data, taxonomy, and phylogenetic tree into a DESeq object and performed a differential ASV abundance analysis using the *DESeq2* v1.36.0 package (Love et al., 2014; McMurdie and Holmes, 2014). This method accounts for potential limitations associated with the use of rarefied microbial community data for the analysis of differential abundance (McMurdie and Holmes, 2014).

#### 2.6.2 Alpha diversity

The alpha diversity of the iteratively rarefied microbiota abundances was calculated using Chao1 diversity (estimated species richness), Simpson diversity (commonly occurring or dominant species), and Shannon diversity (community evenness) to characterize multiple aspects of microbial alpha diversity (Kim et al., 2017). The effect of mercury content and sex on alpha diversity was then tested using linear mixed models implemented in *lme4* v1.1–31 (Bates et al., 2015) with mercury content (high vs. low) and sex as fixed factors and host population as a random factor. Statistical inference of the three models was assessed using Type III Wald F-tests summarized in Analysis of Deviance tables using the *car* v3.1–1 package (Fox and Weisberg, 2019). To further describe differences in alpha diversity between host populations, a one-way ANOVA on each of the indices with population as a main effect was conducted. Lastly, significant pairwise differences between individual factor levels were identified by performing *post hoc* analyses with Tukey’s ‘Honest Significant Difference’ method on all models with significant effects.

#### 2.6.3 Beta diversity

To compare the overall community composition of the stickleback gut microbiota among individuals and host populations, beta diversity was estimated using both quantitative and qualitative measures for both phylogenetic and non-phylogenetic dissimilarity metrics. Quantitative measures (e.g., Bray-Curtis & weighted UniFrac) are weighted by the relative abundance of a species and are thus less sensitive to rare ASVs, whereas qualitative measures (e.g., Jaccard & unweighted UniFrac) are based on species presence/absence and are thus less sensitive to highly abundant ASVs (Lozupone et al., 2007). Therefore, both Bray-Curtis and Jaccard distance matrices were calculated. Then, to also consider the phylogenetic similarity/dissimilarity of ASVs, unweighted UniFrac and weighted UniFrac distance matrices were created using the *rbiom* v1.0.3 (Smith, 2021) package (Lozupone et al., 2007; Lozupone and Knight, 2005). These distance matrices of bacterial communities were then visualized using non-metric multidimensional scaling (NMDS) ordinations, and the first (NMDS1) and second (NMDS2) dimensions were compared between stickleback populations with high and low mercury accumulation levels. Furthermore, a permutational analysis of variance (PERMANOVA) was performed (10,000 permutations) on the dissimilarity matrices to quantify the effect of sex and mercury accumulation on gut microbial community composition. This was done while taking a random nested host population effect into account by restricting permutations to blocks of populations with high and low mercury accumulation.

## 3 Results

### 3.1 Sequencing and ASV identification

High-throughput sequencing of the 128 16S rRNA amplicon libraries yielded a total of 4,248,471 raw reads. One library of a host individual from the Abeek (abe) population (76 reads) had to be discarded due to a low read count. After adapter removal, quality trimming, de-noising, and merging of paired-end reads, the remaining 127 libraries yielded a total of 3,169,508 high-quality reads with an average of 24,957 reads per individual (Supplementary Table S2). From these reads, 9,827 different ASVs were identified, of which 573 were removed for being either singletons (*n* = 311), unassigned ASVs (*n* = 21), or belonging to mitochondria (*n* = 24), chloroplasts (*n* = 199), Archaea (*n* = 19), or Euryarchaeota (*n* = 5). Interestingly, the ASVs assigned as chloroplasts appeared to be most abundant in the Laakbeek (lak) population. However, their inclusion or removal was found not to affect downstream statistical inferences. The quality-trimmed and ASV-filtered libraries had a total read count ranging between 5,206 and 47,217, which equated to a 9-fold difference in sampling depth (Supplementary Table S2). Rarefaction to an even depth of 9,500 reads averaged over 10,000 iterations effectively eliminated this difference but necessitated the removal of 6 samples (abe: *n* = 2; lak: *n* = 2; mom: *n* = 2) with low sequencing coverage. Retaining these individuals would have necessitated rarefaction to 5,000 reads, which would have greatly reduced the sampling depth for individuals with higher read counts. A total of 8,843 different ASVs remained after rarefaction to 9,500 reads (Supplementary Table S2).

### 3.2 Taxon composition

**Figure 2:**
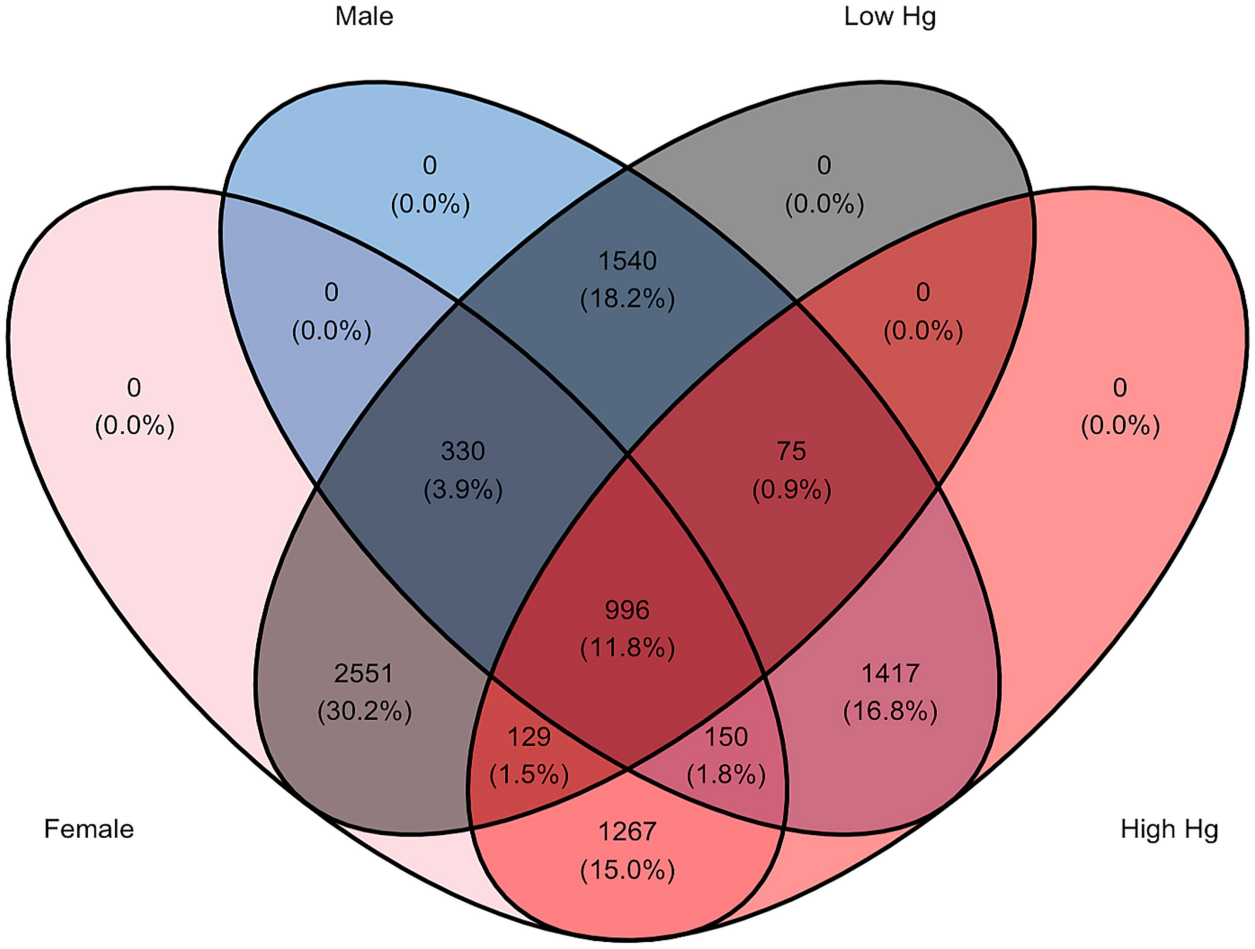
Venn diagram of the distribution of the total number of amplicon sequence variants (ASVs) across the high-low mercury (red and black, respectively) and male–female (blue and pink, respectively) categories.

The gut microbiota of fish with high and low levels of mercury accumulation consisted of a mix of shared and unique ASVs (Figure 2). Three-spined stickleback with a high muscle mercury content hosted 2,834 unique ASVs and shared 1,200 ASVs with hosts with low mercury accumulation. The latter group hosted a 55.9% greater number of unique ASVs (i.e., 4,421), despite the lower number of stickleback individuals in this group (57 vs. 64; Supplementary Table S3; Figure 2). The proportion of all ASVs unique to any level of mercury content (high or low) was thus 6-fold greater than the proportion shared across host populations with high and low muscle mercury content. There also appeared to be a difference in the mix of shared and unique ASVs across male and female individuals. Of the 4,421 ASVs unique to hosts with low mercury content, 2,551 (30.2% of total ASVs) were unique to females, 1,540 (18.2% of total ASVs) were unique to males, and a mere 330 (3.9% of total ASVs) were shared among the sexes (Figure 2). Similarly, of the 2,834 ASVs unique to hosts with high muscle mercury content, 1,267 (15.0% of total ASVs) were unique to females, 1,417 (16.8% of total ASVs) were unique to males, and 150 (1.8% of total ASVs) were shared among sexes (Figure 2). In other words, the number of ASVs unique to both the mercury content level and sex was between 4.5 to 9.5-fold greater than the number of ASVs unique to the mercury content level but shared among sexes. Conversely, of the 950 ASVs that were shared between individuals with low and high levels of mercury content, the majority (788 ASVs or 14.8% of total ASVs) were also shared across the sexes (Figure 2). The overall sex ratios (female: male) for populations with high and low mercury content were comparable (1: 0.84, SD = 0.2 and 1: 1.13, SD = 1.8, respectively; Supplementary Table S3).

**Figure 3:**
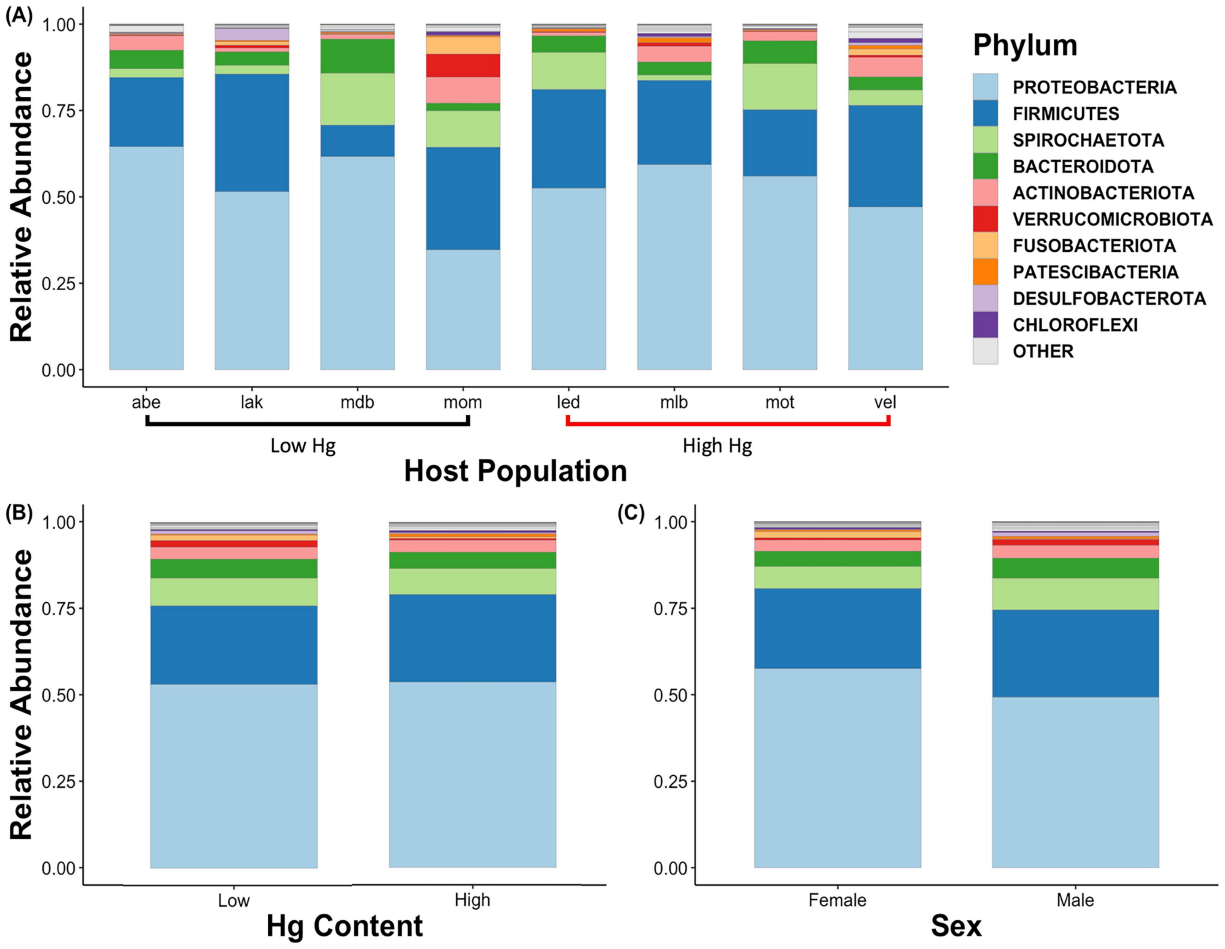
Bar plots of the phylum-level community structure of the three-spined stickleback’s gut microbiota. Community structure is represented by the relative abundances of the 10 most abundant phyla and summarized for each of **(A)** the four host populations with low mercury accumulation and four host populations with high mercury accumulation, **(B)** low and high levels of accumulated mercury, and **(C)** sexes.

**Figure 4:**
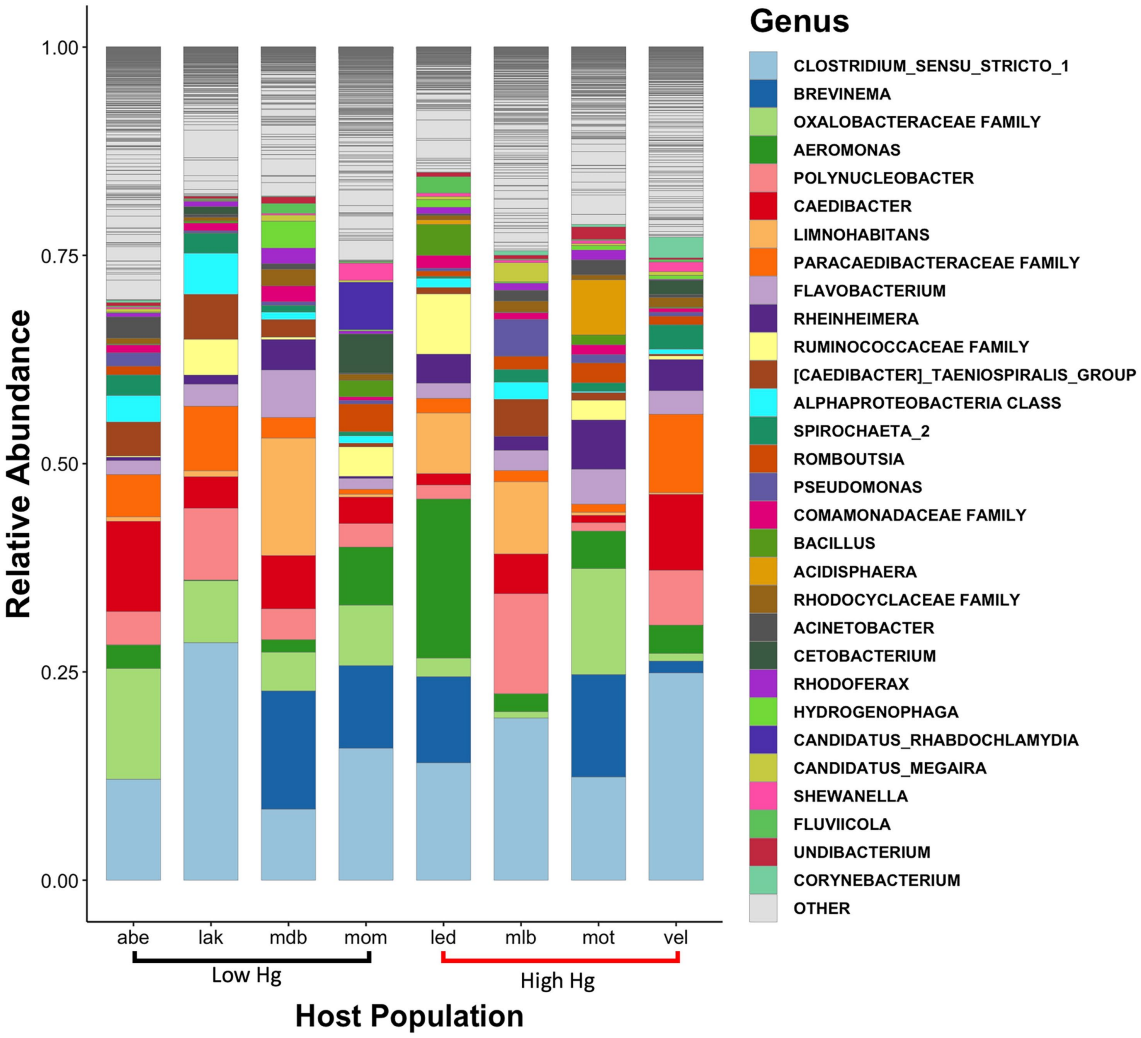
Bar plot of the genus-level community structure of the three-spined stickleback gut microbiota. Community structure is represented by the relative abundances of the 30 most abundant genera and summarized for each of the eight host populations with low (black) and high (red) levels of mercury accumulation.

From these 8,843 ASVs, a total of 41 phyla were detected. Of these, the two most abundant phyla accounted for about 60 to 80% of the relative abundance of these gut microbiota irrespective of host population, mercury content level, or sex (77.7% read total; Figure 3). The two most abundant phyla were Proteobacteria (53.5% of read total) and Firmicutes (24.17% of read total; Figure 3). These phyla were, in turn, dominated by the classes Gammaproteobacteria (38.0% of read total), Clostridia (21.9%), and Alphaproteobacteria (15.5% of read total). The most common genera were *Clostridium sensu stricto 1*, *Brevinema, Aeromonas, Polynucleobacter,* and *Caedibacter* (Figure 4).

**Figure 5:**
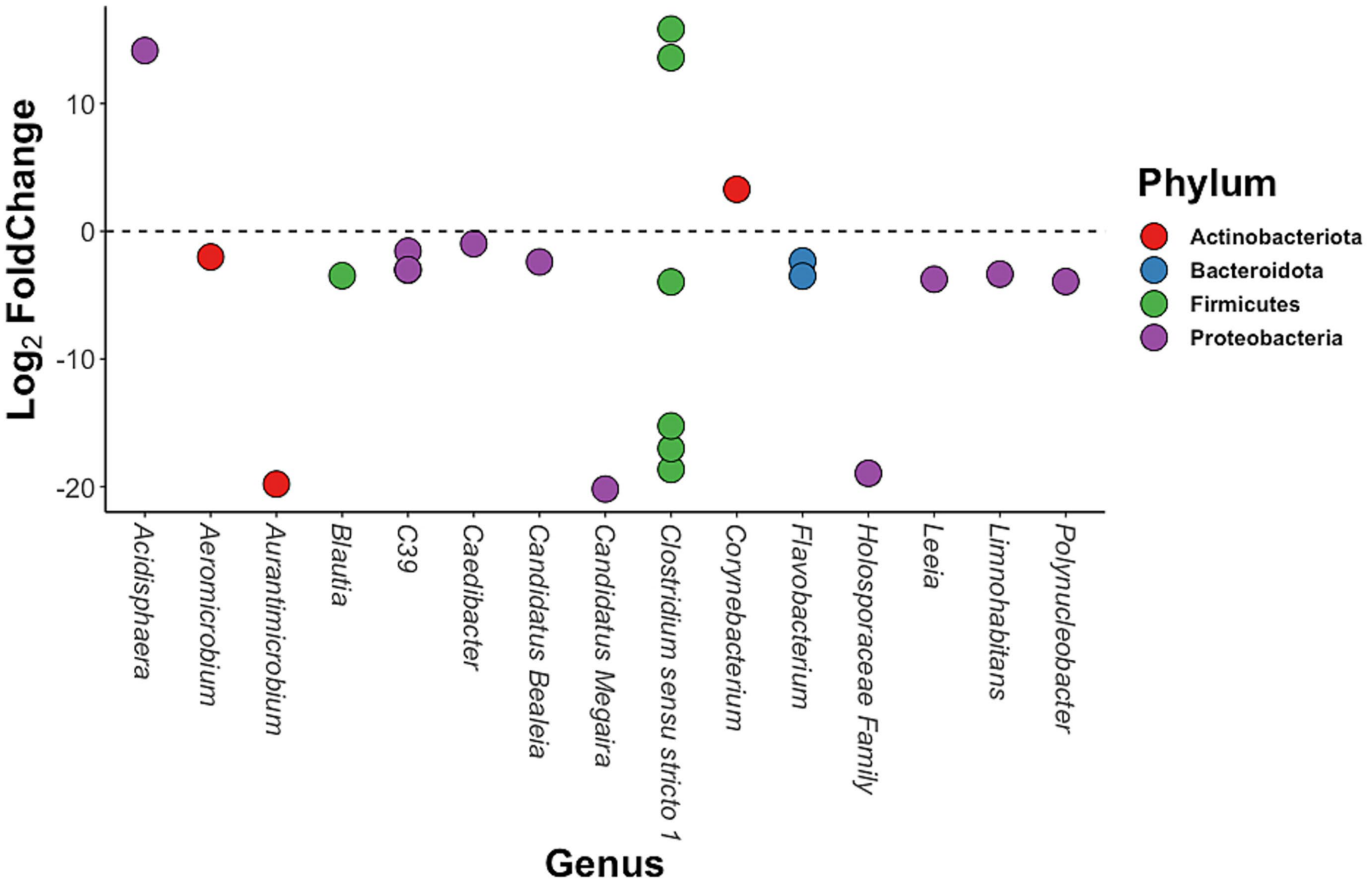
Plot of the 22 differentially abundant ASVs in three-spined stickleback hosts with high mercury accumulation. Differentially abundant ASVs in individuals with a high muscle mercury content compared to those with a low muscle mercury content (i.e., ASVs with |Log_2_FoldChange| > 0 and Benjamini-Hochberg corrected *p*-value < 0.01) are plotted by their corresponding genus and colored according to their phylum. One ASV of an unidentified genus was assigned at the family level to Holosporaceae.

The abundances of 22 bacterial ASVs assigned to 14 different genera and across four phyla were significantly different between host populations with low and high mercury accumulation (Figure 5). Of these, merely four ASVs were significantly more abundant in individuals with high mercury accumulation, whereas 18 ASVs were significantly less abundant (Figure 5). Proteobacteria had near-exclusively negative Log_2_ Fold Change values and represented 9 out of 18 significantly less abundant ASVs (Figure 5). An ASV assigned to the genus *Acidisphaera* was the only member of the Proteobacteria phylum with a significant increase in abundance (Log_2_FoldChange > 200-fold; Figure 5). The genera *Candidatus Megaira* (Log_2_ Fold Change of −20.19), *Aurantimicrobium* (Log_2_ Fold Change of −19.79), and an unidentified genus belonging to the Holosporaceae family (Log_2_ Fold Change of −18.95) had ASVs with the greatest reduction in abundance in high mercury individuals (Figure 5). In addition, three ASVs of the genus *C39* (family: Rhodocyclaceae) were significantly less abundant in high mercury individuals (Figure 5). Interestingly, one of these three ASVs was also the only significant indicator identified by the Dufrene-Legendre indicator analysis for the low mercury accumulation condition. No significant indicators were found for the high mercury condition. Notably, ASVs of the genus *Clostridium sensu stricto 1* ranged between a Log_2_ Fold Change of −17.01 and 15.82, meaning that the abundance of some ASVs of this genus was enriched, whereas others were greatly reduced (Figure 5).

### 3.3 Alpha diversity

**Figure 6:**
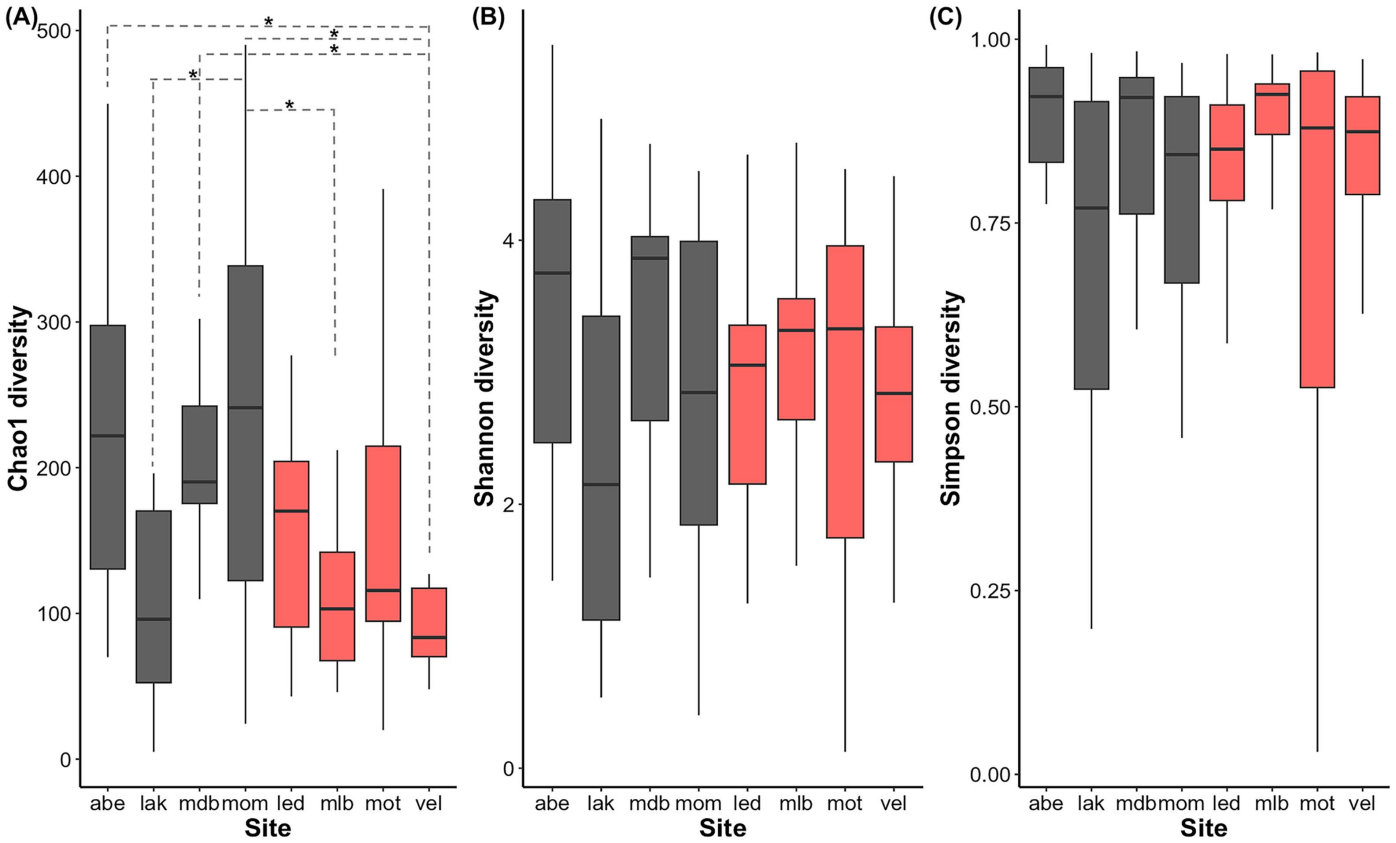
Box plots of the alpha diversity of three-spined stickleback gut microbiota by host population. **(A)** Chao1 diversity, **(B)** Shannon diversity, and **(C)** Simpson’s diversity of the iteratively rarefied (10,000 iterations) bacterial ASV abundances of three-spined stickleback individuals with a high (red) and low (black) muscle mercury content. The bold horizontal bars correspond to the medians, the lower and upper hinges to the first and third quartiles, and whiskers extend to the most extreme value within 1.5*IQR (interquartile range). Significant pairwise differences (P_HSD_ < 0.05) in alpha diversity between host populations are indicated with dashed lines and asterisks.

When accounting for the random host population effect, the alpha diversity of the gut microbial communities neither varied significantly between males and females nor between the high and low mercury populations (Chao1: *p*-value > 0.05; Shannon: *p*-value > 0.05; Simpson: *p*-value > 0.05; Figure 6). Furthermore, gut microbial alpha diversity only differed across the eight host populations for estimated species richness (Chao1 diversity: Adj. *R*^2^ = 0.17, *p*-value < 0.001; Figure 6) but not for Shannon diversity or Simpson diversity (*p*-value > 0.05).

### 3.4 Beta diversity

Host populations with a high and low mercury content showed several differences in gut microbiome composition. Permutational ANOVAs comparing the beta diversity matrices of the iteratively rarefied bacterial ASV abundances across mercury content (high vs. low), sex, and host population demonstrated that host population nested in mercury content level explained the most variation in gut microbial community composition (Bray-Curtis: *R*^2^ = 0.163, *p*-value < 0.0001; Jaccard: *R*^2^ = 0.121, *p*-value < 0.0001; unweighted UniFrac: *R*^2^ = 0.173, *p*-value < 0.0001; weighted Unifrac: *R*^2^ = 0.142, *p*-value < 0.0001; Table 1). The level of mercury accumulation (high vs. low) in the muscle of the stickleback individuals itself explained 1.5 to 2.4% of the variation in gut microbial beta diversity (Bray-Curtis: *R*^2^ = 0. 020, *p*-value < 0.0001; Jaccard: *R*^2^ = 0. 015, *p*-value < 0.0001; unweighted UniFrac: *R*^2^ = 0. 024, *p*-value < 0.0001; weighted Unifrac: *R*^2^ = 0. 011, *p*-value < 0.0001; Table 1). PERMANOVAs of two distance metrics also indicated that sex explained about 1% of the variation in gut microbial community composition (Bray Curtis: *R*^2^ = 0.013, *p*-value = 0.0051; Jaccard: *R*^2^ = 0.012, *p*-value = 0.0075), whereas both distance metrics accounting for phylogenetic distance did not reveal a significant difference in beta diversity between sexes (Table 1). However, between 5 and 7% of the variation in the Bray-Curtis, Jaccard, and weighted UniFrac distances was explained by an interaction between sex and host population nested in mercury content (Bray Curtis: *R*^2^ = 0.053, *p*-value = 0.0197; Jaccard: *R*^2^ = 0.054, *p*-value = 0.0093; weighted UniFrac: *R*^2^ = 0.070, *p*-value = 0.010; Table 1). In other words, the differences in gut microbial community beta diversity between sexes was host-population dependent.

**Table 1:** Permutational ANOVA results (10,000 permutations). The proportion of variance in the Bray-Curtis, Jaccard, unweighted UniFrac, and weighted UniFrac distances of the iteratively rarefied (10,000 iterations) bacterial ASV abundances explained by mercury content, sex, population nested in mercury content, and two-way interactions. Significant *p*-values are marked in bold.

Distance metric	Term	DF	*R* ^2^	*F*	*P*-value
Bray-Curtis	*Mercury content*	1	0.020	2.751	**<0.0001**
	*Sex*	1	0.013	1.884	**0.0051**
	*Population (Mercury content)*	6	0.163	3.821	**<0.0001**
	Sex * Mercury content	1	0.005	0.701	0.9260
	Sex * Population (Mercury content)	6	0.053	1.239	**0.0197**
	*Residual*	–	0.746	–	–
Jaccard	*Mercury content*	1	0.015	2.055	**<0.0001**
	*Sex*	1	0.012	1.581	**0.0075**
	*Population (Mercury content)*	6	0.121	2.671	**<0.0001**
	Sex * Mercury content	1	0.006	0.831	0.8567
	Sex * Population (Mercury content)	6	0.054	1.192	**0.0093**
	*Residual*	–	0.792	–	–
Unweighted UniFrac	*Mercury content*	1	0.024	3.406	**<0.0001**
	*Sex*	1	0.006	0.894	0.5183
	*Population (Mercury content)*	6	0.173	4.038	**<0.001**
	Sex * Mercury content	1	0.006	0.777	0.6932
	Sex * Population (Mercury content)	6	0.044	1.020	0.4027
	*Residual*	–	0.748	–	–
Weighted UniFrac	*Mercury content*	1	0.011	1.573	**<0.0001**
	*Sex*	1	0.007	1.020	0.3940
	*Population (Mercury content)*	6	0.142	3.228	**<0.0001**
	Sex * Mercury content	1	0.003	0.377	0.9762
	Sex * Population (Mercury content)	6	0.070	1.586	**0.0010**
	*Residual*	–	0.767	–	–

**Figure 7:**
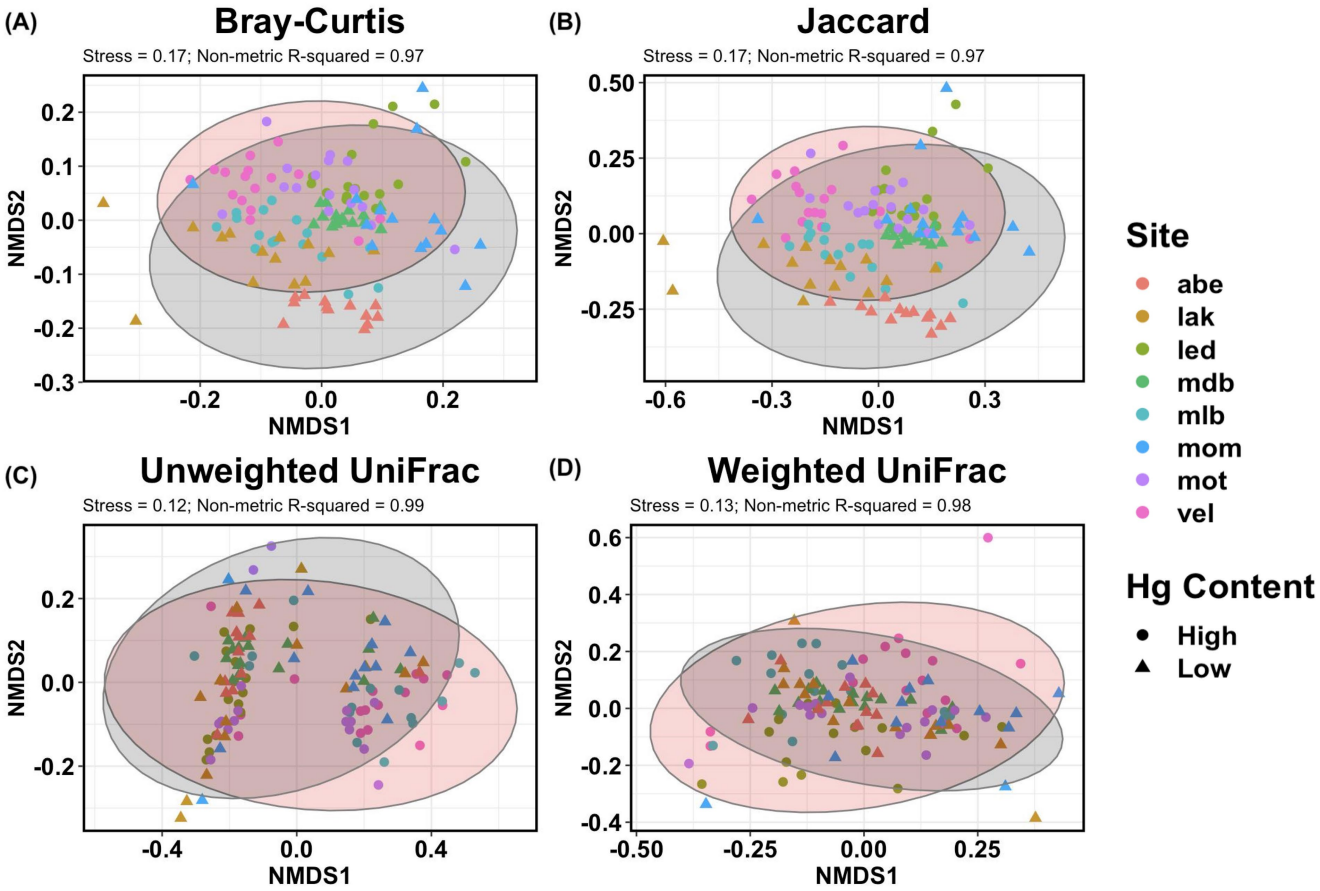
Non-metric multidimensional scaling (NMDS) ordinations of the beta diversity of the gut microbiota of three-spined stickleback. Beta diversity is estimated with NMDS ordinations of **(A)** abundance-weighted distances (Bray-Curtis; stress = 0.17, *R*^2^ = 0.97), **(B)** presence/absence-based distances (Jaccard; stress = 0.17, *R*^2^ = 0.97), **(C)** phylogenetically informed presence/absence-based distances (unweighted UniFrac; stress = 0.12, *R*^2^ = 0.99), and **(D)** phylogenetically informed abundance-weighted distances (weighted UniFrac; stress = 0.13, *R*^2^ = 0.98) of the ASVs abundances rarefied to an equal sampling depth of 9,500 (averaged over 10,000 iterations). Points represent the individual gut microbial communities, which are colored by the host population. Circles and triangles mark host individuals from populations with high and low average muscle mercury accumulation, respectively.

NMDS ordinations further confirmed that the gut microbial communities clustered primarily by host population (Figure 7). Although clustering by total mercury content was more diffuse than by host population, there was some separation between the bacterial communities of host populations with a high and low mercury content (Figure 7). Following Clarke’s (1993) rule of thumb for interpretation of stress values derived from fitting ecological data, current stress values (ranging from 0.12 to 0.17) suggest a usable fit.

## 4 Discussion

To test for the effect of mercury on the diversity and composition of the gut microbiome in three-spined stickleback, we characterized the gut microbiota of four stickleback populations with high- and four populations with low levels of mercury accumulation. The median mercury accumulation levels of these two sets of populations were above and below the European Biota Quality Standard of 0.020 mg kg^−1^ wet weight, respectively. While gut microbial communities were clearly population-specific, the variation in microbial community composition was also associated with host sex, as well as with the level of mercury accumulation. In the next paragraphs, we discuss the variation in community composition in terms of taxon composition, alpha diversity and beta diversity.

### 4.1 Taxon composition

Overall, Proteobacteria and Firmicutes dominated the gut microbiota of the three-spined stickleback irrespective of host population, sex, and muscle mercury concentration. The dominance of these phyla is commonly reported in stickleback and other fish species (Abdelhafiz et al., 2022; Bolnick et al., 2014; Gupta et al., 2019; Shankregowda et al., 2023). Based on the relative abundances of the ten most common phyla, the composition of the gut microbiome differed primarily between host populations. Yet, our analyses also revealed differences in gut microbial composition between sexes, as the proportion of ASVs shared between males and females (17.5% of total ASVs) was substantially smaller than the proportion unique to each sex (82.5% of total ASVs). Likewise, the proportion of ASVs shared between host populations with high and low mercury levels (14.2% of total ASVs) was substantially smaller than the proportion unique to each level (85.8% of total ASVs).

The role and ecological niche of various ASVs that contributed to the differences in taxon composition of gut microbiota between populations with contrasting mercury levels have been reported in previous studies. First, we observed strong differences in the abundance of multiple ASVs identified as Clostridia (Phylum: Firmicutes). Clostridia ssp. commonly occur in the microbial communities of fish gastrointestinal tracts (e.g., Huang et al., 2020; Xu et al., 2022; Lilli et al., 2023). Although pathogenic species exist, Clostridia mostly have commensal relationships with their hosts and are strongly involved in modulating gut homeostasis (Lopetuso et al., 2013). Moreover, Fuess et al. (2021) revealed positive correlations between the abundance of Clostridiaceae taxa and the expression of immune genes in three-spined stickleback. Clostridia ssp. have also been identified as substantial contributors to the active fraction of the gut microbiota in response to xenobiotics (Maurice et al., 2013). We found that the abundance of four ASVs of the genus *Clostridium sensu stricto 1* were significantly reduced in individuals with high muscle mercury content, whereas the abundance of two others were significantly enhanced. The reported shifts in the abundance of these ASVs suggest an active role played by Clostridia ssp. in response to mercury as a xenobiotic stressor in the gut ecosystem. We furthermore identified the *Blautia* genus (class: Clostridia) among the ASVs that were reduced in our host populations with high muscle mercury concentrations. This genus is commonly found in fecal and intestinal samples and has probiotic effects in mammalian hosts (Liu et al., 2021). Their reduction in stickleback hosts with elevated levels of mercury accumulation may thus indicate deleterious changes in the functional composition of their gut microbiota.

Second, we identified 18 ASVs with reduced abundance in the high mercury populations. Among these, two ASVs of the genera *Aurantimicrobia* and *Candidatus Megaira*, as well as one ASV of an unidentified genus of the Holosporaceae family had the greatest reduction in abundance. The genus *Ca. Megaira* and the family Holosporaceae both consist of gram-negative bacteria of the order Rickettsiales, which are obligate endosymbionts commonly occurring in aquatic ciliated protists and—in the case of *Ca. Megaira—*in green algae, amoeba, protozoa, and other protists (Lanzoni et al., 2019; Santos and Massard, 2014). Lanzoni et al. (2019) argue that the retrieval of *Ca. Megaira* from samples such as the current intestinal samples is likely uninformative of their association with the host. *Aurantimicrobia,* on the other hand, is contained in the Microbacteriaceae family. The only reported member in the genus (*A. minutum*) was isolated from river water and exists under non-saline conditions at pH 7 to pH 9 (Nakai et al., 2015). Although the presence of this ASV in the gut microbiota may originate from the host’s aquatic environment, the exact origins of this ASV cannot be determined within the bounds of the current study.

Third, we found a reduction in two ASVs of genus *C39* (family: Rhodocyclaceae) in host populations with high muscle mercury levels. One of these ASVs was also identified by our Defrene-Legendre indicator species analysis as a significant indicator of the low mercury level condition. Although *C39* is predominantly detected in freshwater microbiota (e.g., Cannon et al., 2017; Wang et al., 2022; Zhou et al., 2017; Zhu et al., 2022), it has also been detected in gut microbial samples of larval bush mosquito (*Aedes koreicus*; Alfano et al., 2019). Similarly, the composition of fish gut microbiota tends to be strongly influenced by the bacterial communities of the surrounding aquatic environment (Chen et al., 2022; Giatsis et al., 2015). It is thus plausible that the stickleback gut microbiota is, in part, derived from the aquatic environment. Interestingly, in heavily polluted river water, Zhu et al. (2022) reported a positive correlation between *C39* abundance and water quality and designated it as a biomarker for water quality. Other studies on aquatic microbiota found significant shifts in its abundance in the presence of anthropogenic pollutants such as heavy metals (Liguori et al., 2021) and urban wastewater (Wang et al., 2022). The reduction or even absence of *C39* in host populations with high mercury levels could thus reflect differences in quality of the environmental conditions of the host’s aquatic environment.

Finally, the abundance of four ASVs was enhanced in the high mercury populations. Among those, the abundance of an ASV of the genus *Acidispaera* was most enhanced. This genus currently contains a single species (*A. rubrifaciens*) which is an obligate acidophile that occurs in acidic hot springs and acidic mine drainage and can grow between pH 3.5 and pH 6.0 (Hiraishi et al., 2000). All water pH measured at our sampling sites exceeded 7.0, making this identification rather unlikely. However, several genera that are closely related to *Acidispaera* (e.g., *Acidophilium* and *Rhodopila*) also preferentially occur under the anoxic and acidic conditions that are more conducive for methylmercury production (Hiraishi et al., 2000; Imhoff and Madigan, 2021; Ullrich et al., 2001). However, the reason for the enhancement of this ASV in host populations with high mercury accumulation remains uncertain.

### 4.2 Alpha diversity

Alpha diversity indices neither indicated any differences between sexes, nor high and low mercury content. Alpha diversity did not differ between host populations either, except for population-related differences in Chao1 diversity. This is surprising since differences in gut community alpha diversity have been reported across comparable geographic scales in populations of three-spined stickleback (Shankregowda et al., 2023; Smith et al., 2015). Moreover, exposure to pollutants has previously been associated with shifts in the alpha diversity of microbiota. For instance, Watson et al. (2021) reported a significant negative association between gut microbial alpha diversity and mercury concentrations in the hair of polar bears. Similarly, Fackelmann et al. (2023) demonstrated positive correlations between the accumulation of microplastics and gut microbial alpha diversity in wild seabirds. Conversely, our results indicate that the alpha diversity of the gut microbial communities appears relatively stable across the three-spined stickleback populations inhabiting the Flemish riverscape. This indicates that population-, sex-, or pollution-associated differences in gut microbiota can manifest in various ways, reiterating the importance of investigating multiple aspects of microbial community diversity and composition.

### 4.3 Beta diversity

We consistently found a significant association between beta diversity and the level of mercury accumulation in the host population, which explained up to 2.4% of the variance in gut community composition. This association also remained significant when accounting for the taxonomic relatedness and abundance of ASVs. Although this may be a relatively small effect size, it is in the same range as the effect size reported in a study investigating the effect of metallic micropollutants on the gut microbiota of wild European eel (*Anguilla anguilla*) populations (Bertucci et al., 2022). Additionally, an association between mercury exposure and beta diversity has been demonstrated experimentally. Zhao et al. (2020) demonstrated that controlled oral administration of methylmercury in laboratory mice caused intestinal injuries and major genus-level shifts in the gut microbiome, and they reported a significant correlation between the compositional shifts in the gut microbiota and the expression of apoptotic genes in the gut. Although the current results do not allow for such identifications of functional impairment, they do suggest that the composition of commensal microbial communities in the stickleback intestinal tract is affected by this environmental contaminant.

Our results further indicated that beta diversity was significantly associated with population-specific factors. Indeed, gut microbial communities clustered predominantly by the sampling location of the corresponding host population. This association accounted for approximately 15% of the variance in overall bacterial community composition. The composition of gut microbial communities is known to be affected by numerous factors specific to the host environment and may show substantial variation at geographic scales as well as at finer spatial scales (Goertz et al., 2019; Nobles and Jackson, 2020). Food web structure and diet composition, for example, may vary between sampling locations and can strongly affect mercury exposure and bioaccumulation in fish as well as the composition of their gut microbiota (Bertucci et al., 2022; Bolnick et al., 2014; Johnston et al., 2022; Kahilainen et al., 2016; Power et al., 2002). Although the exact contributions of such underlying drivers remain unclear, the location-specific pattern observed in this study is consistent with findings by Shankregowda et al. (2023) based on other stickleback populations in the Flanders region.

## 5 Conclusion

Our study revealed that the community composition as well as the abundance of several members of the gut microbiota of three-spined stickleback populations in Flanders are associated with mercury accumulation. Yet, we did not find specific indicator species for high mercury levels. Taken together with previous studies conducted on the same populations, we conclude that mercury is a xenobiotic whose presence in natural populations can be related to detectable differences in both the genome and the gut microbiome. However, these differences represent contrasts between natural populations and do not allow us to infer causality. Controlled experiments would be required to conclude whether or not environmentally relevant mercury concentrations lead to gut dysbiosis in these stickleback populations, and if they show signs of adaptation to high environmental mercury levels. Our results thus provide the rationale for further controlled experiments aiming to test for a causal relationship between mercury exposure, shifts in the functional composition of the gut microbiota, and its adaptive implications in three-spined stickleback.

## Data Availability

The datasets presented in this study can be found in online repositories. The names of the repository/repositories and accession number(s) can be found at: https://www.ncbi.nlm.nih.gov/, PRJNA1279194.
