# Supplementary material for: Differential gut microbiome composition in three-spined stickleback populations with contrasting levels of mercury accumulation
Source: Front Microbiol. 2026 Jan 7;16:1673354. doi: 10.3389/fmicb.2025.1673354 (PMC12819629; doi:10.3389/fmicb.2025.1673354)
Supplement: Supplementary file 1 [file Data_Sheet_1.pdf]

Supplementary Material

Differential Gut Microbiome Composition in Three-Spined Stickleback Populations with Contrasting Levels of Mercury Accumulation

Marijn Kuizenga [1*], Aruna M. Shankregowda [1,2], Prabhugouda Siriyappagouder [1], Vyshal Delahaut [3], Federico C.F. Calboli [4,5], Lieven Bervoets [3], Brijesh Singh Yadav[1], Filip A.M. Volckaert [4], Gudrun De Boeck [3], Joost A.M. Raeymaekers[1*]

[1] Faculty of Biosciences and Aquaculture, Nord University, N-8049 Bodø, Norway

[2] Department of Biosciences, Swansea University, Swansea SA2 8PP, United Kingdom

[3] ECOSPHERE, Department of Biology, University of Antwerp, Groenenborgerlaan 171, B-2020 Antwerp, Belgium

[4] KU Leuven, Laboratory of Biodiversity and Evolutionary Genomics, Ch. Deberiotstraat 32, B- 3000 Leuven, Belgium

[5] Natural Resources Institute Finland (Luke), Latokartanonkaari 9, 00790 Helsinki, Finland

(*) Correspondence: marijnkuizenga@me.com, joost.raeymaekers@nord.no

Table S1. Geographic characteristics and mercury measurements of 21 three-spined stickleback populations in Flanders (Belgium). Geographic characteristics include basin (Maas, Scheldt-West (SW), Scheldt-East (SE)), river, code, longitude (WGS84LONG) and latitude (WGS84LAT). Mercury measurements include total mean mercury concentration in muscle tissue (mean Hg; $ng.g^{-1}$ dry weight; SD: standard deviation), total median mercury concentration in muscle tissue (median Hg; $ng.g^{-1}$ dry weight), and mercury concentration measured in the sediment (Hgsed; $\mu g.kg^{-1}$ dry solids). Locations are sorted by median Hg. All data were obtained by Calboli et al. (2021). For the present study, we selected the eight locations marked in grey, including the four locations with the lowest median mercury concentration in muscle tissue (abe, mdb, mom, lak), as well as the four locations with the highest median mercury concentration in muscle tissue (vel, mot, led, mlb).

NR	Basin	River	Code	WGS84LONG	WGS84LAT	mean Hg ± SD	median Hg	Hgsed
8	Maas	Abeek	abe	5.513423	51.10708	22.04 ± 13.93	21.56	13.18
21	SW	Molenaarsdreefbeek	mdb	4.004735	51.16604	49.89 ± 20.89	45.13	113.40
12	SE	Mombeek	mom	5.286693	51.08485	102.41 ± 127.89	47.50	156.34
10	SE	Laakbeek	lak	4.847037	51.23132	55.12 ± 29.15	50.98	158.39
5	Maas	Jeker	jek	5.670649	50.81574	89.77 ± 94.17	55.86	208.69
4	Maas	Bosbeek	bos	5.757784	51.09350	89.20 ± 62.11	76.96	59.13
14	SE	Molse Nete	mne	5.003755	51.14764	85.05 ± 52.27	77.80	166.24
7	Maas	Erkbeek	erk	5.555096	51.26707	78.81 ± 51.25	79.66	21.72
3	Maas	Itterbeek	itt	5.643896	51.11733	81.50 ± 50.44	87.87	34.02
17	SW	Meirebeek	mei	3.594099	51.05471	112.02 ± 62.91	94.85	67.04
16	SW	Waalshoekbeek	waa	3.375900	50.89580	127.74 ± 40.60	112.15	282.91
11	SE	Winge	win	4.831537	50.93577	126.08 ± 26.93	123.22	32.82
6	Maas	Dommel	dom	5.428281	51.26934	137.31 ± 70.72	143.55	68.13
19	SW	Oude Schelde	osc	3.923661	51.02479	173.88 ± 50.06	156.03	569.92
13	SE	Voorste Nete	vne	5.114041	51.23171	160.93 ± 71.83	159.56	17.20
2	Maas	Witbeek	wit	5.694276	51.08615	164.25 ± 79.84	163.56	24.59
15	SW	Geluwebeek	gel	3.091013	50.80807	191.40 ± 55.84	178.46	228.31
1	SE	Velpe	vel	4.987955	50.87657	202.48 ± 89.18	195.71	50.14
18	SW	Motebeek	mot	3.413861	51.05757	224.28 ± 69.97	218.23	84.35
20	SW	Lede	led	3.973861	51.10103	321.91 ± 179.95	302.70	96.15
9	SE	Molenbeek	mlb	4.583782	50.92556	326.11 ± 77.77	326.63	174.73

Table S2. The number of samples, sampling depth, and total number of ASVs prior to and after quality trimming, ASV filtering, and iterative rarefaction.

	**Raw Data**	**Quality Trimmed**	**ASV Filtered**	**Rarefied**
**N Total**	128	127	127	121
N Low Hg	64	63	63	57
N High Hg	64	64	64	64
**Total Reads**	4,248,471	3,169,508	3,037,821	1,159,028*
Min.	76	6,158	5,206	9,503
Median	32,926	24,392	22,917	9,568
Mean	33,191	24,957	23,920	9,579
SD	10,311	9,102	9,261	52
Max.	58,828	47,605	47,217	9,779
**Total ASVs**	-	9,827	9,254	8,843

* refers to the total number remaining reads in the rarefied communities (9500 reads) averaged over 10,000 iterations.

Table S3. Sample size of three-spined stickleback individuals by muscle Hg level (low and high), host population, and sex (F and M). N, N_F and N_M mark total sample size, number of females and number of males, respectively.

**Muscle Hg level**	**Host population**	**N**	**N_F**	**N_M**	**Sex Ratio (N_F : N_M)**
Low	abe	13	7	6	1 : 0.86
	lak	14	9	5	1 : 0.56
	mdb	16	8	8	1 : 1
	mom	14	7	7	1 : 1
Total (Low)	-	57	31	26	1 : 0.84
High	led	16	10	6	1 : 0.60
	mlb	16	11	5	1 : 0.45
	mot	16	6	10	1 : 1.67
	vel	16	3	13	1 : 4.33
Total (High)	-	64	30	34	1 : 1.13
